# Knockdown of receptor interacting protein 140 (RIP140) alleviated lipopolysaccharide-induced inflammation, apoptosis and permeability in pulmonary microvascular endothelial cells by regulating C-terminal binding protein 2 (CTBP2)

**DOI:** 10.1080/21655979.2022.2031403

**Published:** 2022-02-03

**Authors:** Qizheng Wang, Qiong Wu

**Affiliations:** a Department of Pediatrics, Huai’an Maternal and Child Health Care Hospital, Huai’an, Jiangu, China; b Department of Pediatrics, The People’s Hospital of China Three Gorges University (The First People’s Hospital of Yichang), Yichang, Hubei, China

**Keywords:** Receptor interacting protein 140 (RIP140), lung injury, pulmonary microvascular endothelial cells (PMVECs), C-terminal binding protein 2 (CTBP2)

## Abstract

The main pathological feature of acute lung injury (ALI) is pulmonary edema caused by increased permeability of pulmonary microvascular endothelial cells (PMVECs). LPS was has been confirmed to lead to cell damage and barrier dysfunction in PMVECs. Furthermore, receptor interacting protein 140 (RIP140) was discovered to be increased in LPS-induced human pulmonary microvascular endothelial cells (HPMECs), but the mechanism of RIP140 on LPS-induced HPMECs has not been investigated. In this study, an acute lung injury model was constructed in LPS-induced HPMECs. After RIP140 was downregulated, inflammation, apoptosis and cell permeability levels were detected by RT-qPCR, TUNEL staining and FITC-Dextran, respectively. Western blotting was used to detect the protein levels of related factors. The binding of RIP140 and C-terminal binding protein 2 (CTBP2) was predicted by database and verified by Co-IP. Subsequently, CTBP2 overexpression was transfected into cells and the above experiments were performed again. The results showed that inflammation, apoptosis and permeability levels of LPS-induced HPMECs were remarkably increased compared to the untreated control group. However, these levels were suppressed after RIP140 was silenced compared to the LPS-induced HPMECs group. Notably, the Co-IP study demonstrated that RIP140 and CTBP2 interacted with each other. Moreover, CTBP2 overexpression reversed the inhibitory effects of RIP140 silencing on LPS-induced inflammation, apoptosis and permeability levels in HPMECs. Together, the study found that interference of RIP140 could alleviate LPS-induced inflammation, apoptosis and permeability in HPMECs by regulating CTBP2.

## Graphical Abstract

**Figure:**
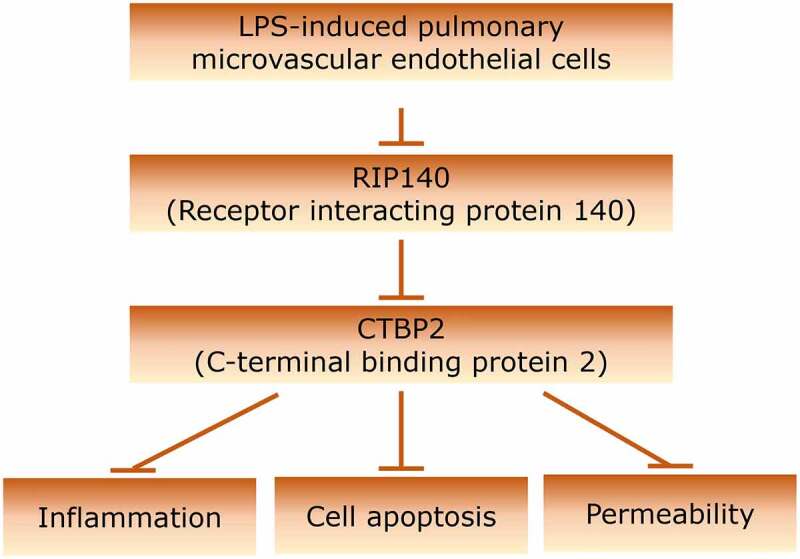

## Introduction

Sepsis acute lung injury is a common clinical critical disease, affecting more than 19 million people worldwide every year, and the in-hospital mortality rate of patients is about 15–25% [1]. Sepsis is also a cause of death in pediatric intensive care units. Globally, it is estimated that there are 2202 cases of sepsis per 100,000 births, with mortality rates in children with sepsis ranging from 4% to as high as 50% [2]. Acute lung injury (ALI) is one of the manifestations of multiple organ dysfunction syndrome caused by sepsis [3]. ALI is characterized by a rapid onset of inflammation and the main pathological feature is pulmonary edema caused by increased permeability of pulmonary microvascular endothelial cells [4–6]. Of concern, previous studies have shown that pediatric intensive care unit admissions are associated with ALI [7,8]. Thus, it is important to develop effective ALI biomarkers to contribute to the diagnosis and treatment of early lung injury in children.

Lipopolysaccharide (LPS) is a major pathogenic mediator of epithelial cell activation which can activate the NF-κB pathway and induce the expression of cell adhesion molecules [9,10]. Hence, LPS is often used to induce lung injury models. It has been suggested that receptor-interacting protein 140 (RIP140, also known as NRIP1) expression is increased in LPS-induced lung tissues and inhibition of RIP140 attenuates LPS-induced release of inflammatory factors from macrophages [11]. Additionally, the study showed that RIP140 expression was increased in LPS-induced lung microvascular cells [12]. However, the mechanism of RIP140 in LPS-induced pulmonary microvascular endothelial cells has not been investigated. The c-terminal binding protein-2 (CTBP2) is a highly conserved transcriptional co-repressor that has been reported to be involved in the development and progression of several human cancers and associated with the poor prognosis of prostate cancer and lung cancer [13–15].

In this study, RIP140 and CTBP2 were found to be correlated by database prediction. Notably, the present study explored for the first time whether RIP140 regulated LPS-induced ALI cell model via CTBP2.

## Materials and methods

### Cell culture and treatment

Human pulmonary microvascular endothelial cells (HPMECs) were obtained from the PromoCell GmBH (Germany). These cells were cultured in Dulbecco’s modified Eagle medium (DMEM) supplemented with 10% fetal bovine serum (FBS) and 1% antibiotics/antimycotics. And these cells were placed in a 37°C humid atmosphere with 5% CO_2_. Cells were induced by 100 ng/ml LPS and incubated at 37°C for 24 h.

### Cell transfection

After 6 h starvation, HPMECs were cultured in 6‑well plates to 70‑80% confluence. si-RIP140 (si-RIP140-1 or si-RIP140-2), siRNA-negative control (si-NC), CTBP2 overexpression plasmid (Ov-CTBP2) and the empty vector (Ov-NC) were synthesized by Shanghai GenePharma Co., Ltd. The transfection was performed using Lipofectamine® 2000 (Invitrogen; Thermo Fisher Scientific, Inc.). Following transfection for 48 h in a humidified atmosphere of 5% CO_2_ at 37°C, cells were harvested for subsequent experiments.

### TUNEL assay

HPMECs (3x10^3^ cells/well) were fixed in 4% formaldehyde at room temperature for 10 min and washed with PBS buffer solution twice. Cells were permeabilized with Triton X-100 in PBS for 20 min and the apoptosis assay was performed using a TUNEL Assay kit (Invitrogen; Thermo Fisher Scientific, Inc.) according to the manufacturer’s protocol. Apoptosis could be observed under a fluorescent microscope (magnification x200; Olympus Corporation).

### Database analysis

The correlation between RIP140 and CTBP2 in lung tissues was predicted using HumanBase Gene Networks database (https://hb.flatironinstitute.org/gene/) and BioGRID database (https://thebiogrid.org/) analysis.

### Co-immunoprecipitation (Co-IP) assay

After the treated HPMECs were harvested, an appropriate amount of cell lysis buffer (including protease inhibitors) was added. The cells were lysed on ice or at 4°C for 30 min, and the supernatant was extracted after centrifugation at 12,000 g for 20 min at 4°C. 1 μg of corresponding antibody and 10 μl protein A/G-beads were added into the remaining lysate, slowly shaken and incubated at 4°C overnight. After immunoprecipitation reaction, protein A/G-beads were centrifuged at 4°C at 3,000 g for 5 min. Protein A/G-beads were centrifuged to the bottom of the tubes. The surplus was removed carefully and protein A/G-beads were washed with 1 ml split buffer 3–4 times. The proteins were eluted by boiling in 1× SDS sample buffer for 10 min and subsequently subjected to Western blotting.

### Detection of endothelial cell permeability

HPMECs were cultured on 0.4-μm transwell inserts. After stimulation, the upper wells were incubated with FITC-dextran (1 mg/ml), and then 50 μl of medium from the bottom chamber was aspirated 30 min later. The absorbance of the medium at the excitation wavelength of 488 nm and the emission wavelength of 520 nm was read with a microplate reader.

### Western blotting

Total protein was extracted from HPMECs using RIPA lysis buffer (Beyotime Institute of Biotechnology) at 4°C for 1 h. Total protein was quantified using a Pierce™ BCA Protein assay kit (Thermo Fisher Scientific, Inc.) and protein (30 μg/lane) was separated via 10% SDS-PAGE and transferred onto PVDF membranes. The membranes were blocked with 5% skim milk for 1 h at room temperature. Subsequently, the membranes were incubated overnight with the following primary antibodies at 4°C: Bcl2 (1:1,000), Bax (1:1,000), cleaved-caspase 3 (1:1,000), CTBP2 (1:1,000) GAPDH (1:2,000). Following, the membranes were washed with TBST and incubated with a horseradish peroxidase-conjugated secondary antibody (1:10,000) for 2 h at room temperature. Proteins bands were visualized using an ECL reagent (Invitrogen; Thermo Fisher Scientific, Inc.). Densitometric analysis was performed using ImageJ software (version 1.49 v; National Institutes of Health).

### Reverse transcription-quantitative PCR (RT-qPCR)

Total RNA was extracted from HPMECs with TRIzol® reagent (Invitrogen; Thermo Fisher Scientific, Inc.). Total RNA was reverse transcribed using RevertAid reverse transcriptase (Invitrogen; Thermo Fisher Scientific, Inc.) at 42°C for 1 h, according to the manufacturer’s protocol. RT-qPCR was performed using the LightCycler 480 SYBR Green I Master kit (Roche Diagnostics) on a LightCycler 480 II (Roche Diagnostics). The following thermocycling conditions were used for qPCR: Initial denaturation at 95°C for 5 min; followed by 45 cycles of amplification, including denaturation at 94°C for 10 sec, annealing at 60°C for 20 sec and a final extension at 72°C for 30 sec.

### Statistical analysis

Statistical analyses were performed using GraphPad Prism version 7.0 software (GraphPad Software, Inc.). Data are presented as the mean ± SD. Statistical differences among groups were determined using a one-way ANOVA followed by a Tukey’s post hoc test. P < 0.05 was considered to indicate a statistically significant difference. All experiments were repeated at least three times.

## Results

### Knockdown of RIP140 reduced LPS-induced inflammatory response in HPMECs

**Figure 1.**
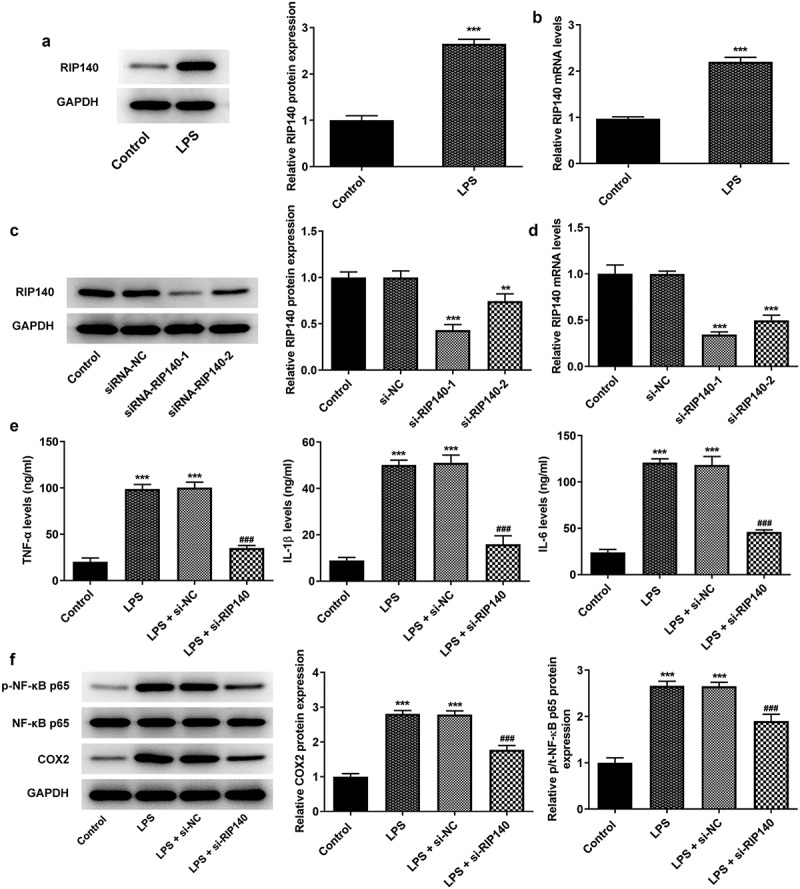
Knockdown of RIP140 attenuates LPS-induced inflammatory response in HPMECs. The protein (a) and mRNA (b) levels of RIP140 in LPS-induced HPMECs. The protein (c) and mRNA (d) levels of RIP140 following transfection of si-RIP140. (e) The expression of TNF-α, IL-1β, and IL-6 was detected by RT-qPCR. (f) The protein levels of COX2, p/t-NF-κB p65 were detected by Western blotting. **P < 0.01, ***P < 0.001 vs. control; ^###^P < 0.001 vs. LPS + si-NC.

The protein and mRNA levels of RIP140 were detected by Western blotting and RT-qPCR, respectively. The results showed that the expression of RIP140 was significantly higher in LPS-induced HPMECs compared with the control group (Figure 1a-1b). After transfection of si-RIP140-1 and si-RIP140-2, the results indicated that RIP140 expression was distinctly cut down and si-RIP140-1 was more effective in RIP140 interference (Figure 1c-1d). Therefore, si-RIP140-1 was used to silence RIP140 in the subsequent experiments (si-RIP140). RT-qPCR analysis revealed that the expression of inflammatory factors including TNF-α, IL-1β and IL-6 was significantly increased in the LPS-induced group compared to the control group (Figure 1e). However, the expression of TNF-α, IL-1β, and IL-6 in the LPS + si-RIP140 group was significantly suppressed compared to the LPS + si-NC group. Similarly, Western blotting analyzed that COX2 and p/t-NF-κB p65 expression was significantly increased following LPS induction while significantly suppressed after RIP140 was silenced compared with the LPS-induced group (figure 1f).

### Knockdown of RIP140 reduced LPS-induced apoptosis and permeability in HPMECs

**Figure 2.**
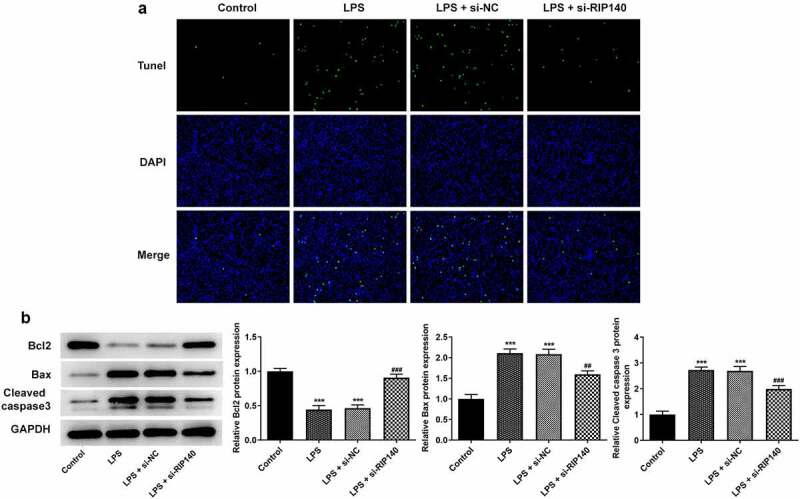
Knockdown of RIP140 inhibits LPS-induced apoptosis in HPMECs. (a) Cell apoptosis was detected by using TUNEL staining. (b) The protein levels of apoptosis-related factors including Bcl2, Bax and cleaved caspase 3 were detected by Western blotting. ***P < 0.001 vs. control; ^##^P *< *0.01 and ^###^P < 0.001 vs. LPS + si-NC.

**Figure 3.**
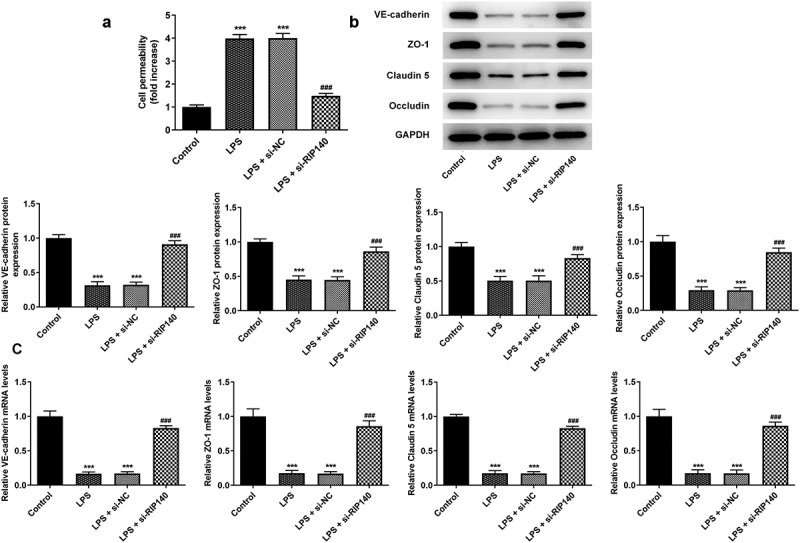
Knockdown of RIP140 reduces LPS-induced permeability in HPMECs. (a) The permeability of HPMECs was detected by FITC-Dextran. The protein (b) and mRNA (c) levels of permeability-related factors including VE-cadherin, ZO-1, Claudin 5 and Occludin. ***P < 0.001 vs. control; ^###^P < 0.001 vs. LPS + si-NC.

Apoptosis was assessed by TUNEL staining and Western blotting. And FITC-dextran and Western blotting were used to detect the permeability of HPMECs and the protein levels of related factors. TUNEL staining demonstrated that induction of LPS increased the apoptosis level of HPMECs compared to the control group. Conversely, the apoptosis of HPMECs was inhibited when RIP140 was downregulated compared to the LPS-induced group (Figure 2a). Meanwhile, detection of apoptosis-related proteins suggested that induction of LPS inhibited the expression of Bcl2 while upregulated the expression of Bax and Cleaved caspase3. Upon RIP140 knockdown, Bcl2 expression was upregulated, and Bax and cleaved caspase3 expression was downregulated compared to the LPS-induced group (Figure 2b). In addition, FITC-dextran assay found that LPS induced a nearly 4-fold increase on the permeability of HPMECs, while this result was restored by interference with RIP140 (Figure 3a). Protein and mRNA levels of VE-cadherin, ZO-1, Claudin5 and Occludin were significantly decreased in the LPS-induced group compared to the control group; however, this effect was reversed in the LPS + si-RIP140 compared to LPS + si-NC group (Figure 3b-3c).

### CTBP2 expression was increased in LPS-induced HPMECs and could bind to RIP140

**Figure 4.**
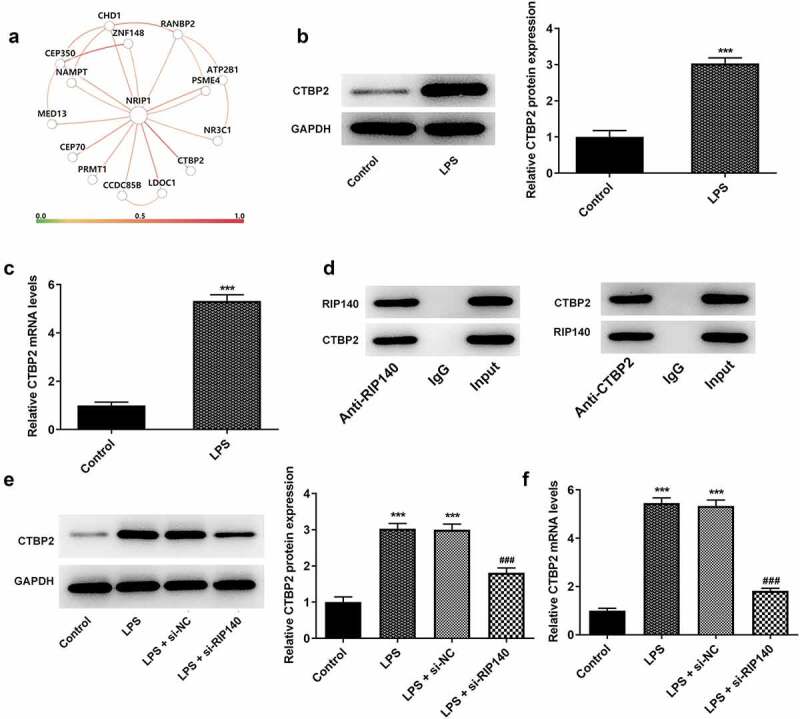
CTBP2 expression is increased in LPS-induced HPMECs and binds to RIP140. (a) The correlation between RIP140 (NRIP1) and CTBP2 in lung tissues was predicted using HumanBase Gene Networks database. The protein (b) and mRNA (c) levels of CTBP2 in LPS-induced HPMECs. (d) The interaction between RIP140 and CTBP2 was detected using Co-IP assay. The protein (e) and mRNA (f) levels of CTBP2 following transfection of si-RIP140. ***P < 0.001 vs. control; ^###^P < 0.001 vs. LPS + si-NC.

The binding relationship between RIP140 and CTBP2 was examined by database analysis, Western blotting and RT-qPCR, respectively. HumanBase Gene Networks database analysis predicted that RIP140 bound to CTBP2 in lung cancer tissues (Figure 4a). Meanwhile, the expression of CTBP2 was significantly higher in LPS-induced HPMECs than in the control group (Figure 4b-4c). The correlation between RIP140 and CTBP2 was further verified by Co-IP assay. The results showed that RIP140 and CTBP2 could bind to each other (Figure 4d). Of interest, the enhanced CTBP2 expression in LPS-exposed HPMECs showed a marked decrease in the LPS + si-RIP140 group compared to the LPS + si-NC group (Figure 4e-4f).

### Overexpression of CTBP2 reversed the suppressive role of RIP140 deficiency in the inflammatory response, apoptosis and permeability in LPS-induced HPMECs

**Figure 5.**
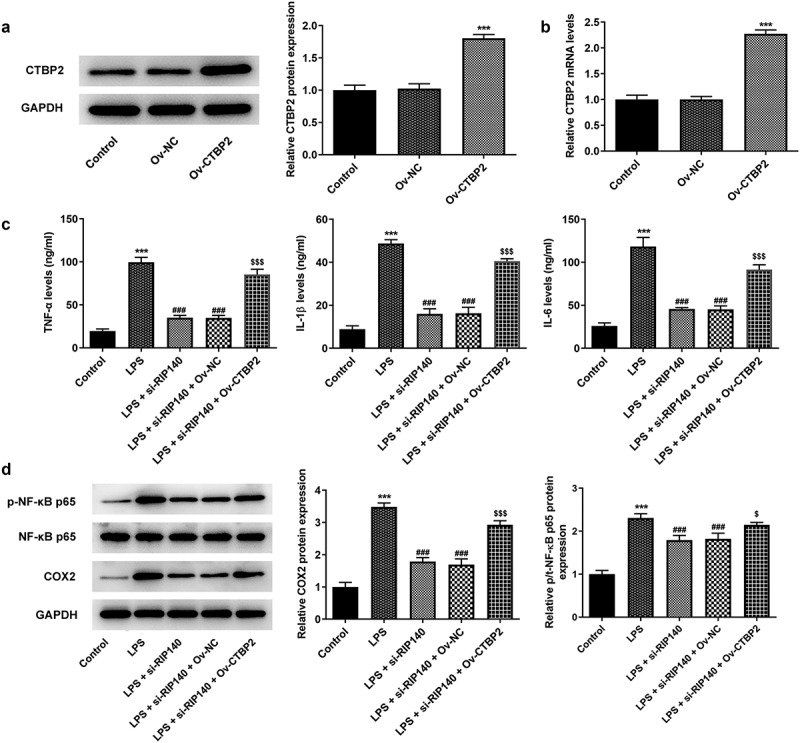
Overexpression of CTBP2 reverses the suppressive role of RIP140 deficiency in the inflammatory response in LPS-induced HPMECs. The protein (a) and mRNA (b) levels of CTBP2 following transfection of Ov-CTBP2. (c) The expression levels of TNF-α, IL-1β, and IL-6 were detected by kits. (d) The protein levels of COX2, p/t-NF-κB p65 were detected by Western blotting. ***P < 0.001 vs. control; ^###^P < 0.001 vs. LPS. ^$^P < 0.05 and ^$$$^P < 0.001 vs. LPS + si-RIP140 + Ov-NC.

**Figure 6.**
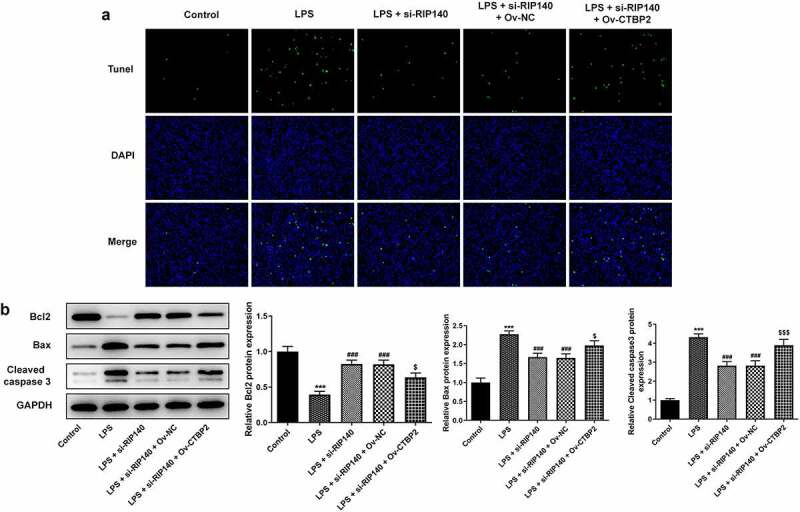
Overexpression of CTBP2 reverses the suppressive role of RIP140 deficiency in the apoptosis in LPS-induced HPMECs. (a) Cell apoptosis was detected by TUNEL staining. (b) The expression of apoptosis-related proteins including Bcl2, Bax and cleaved caspase3 were detected by Western blotting. ***P < 0.001 vs. control; ^###^P < 0.001 vs. LPS. ^$^P < 0.05 and ^$$$^P < 0.001 vs. LPS + si-RIP140 + Ov-NC.

**Figure 7.**
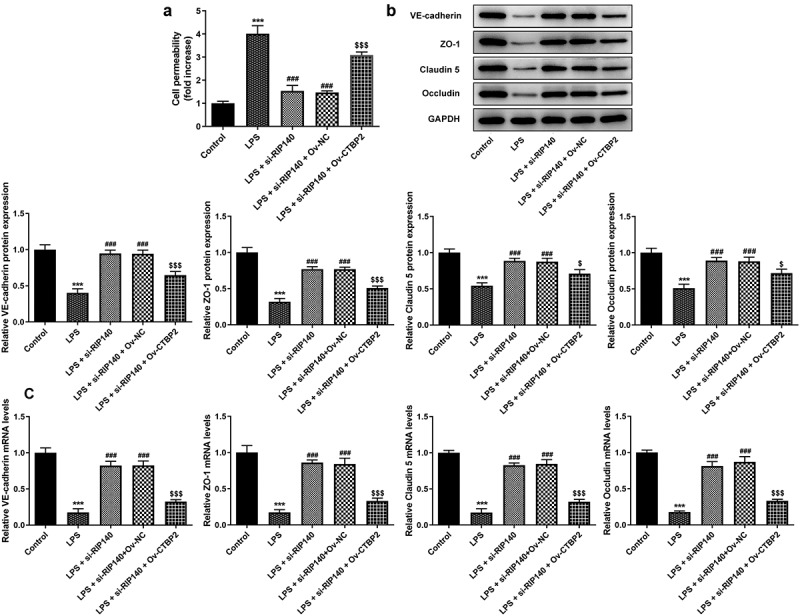
Overexpression of CTBP2 reverses the suppressive role of RIP140 deficiency in the permeability in LPS-induced HPMECs. (a) The permeability of HPMECs was detected by FITC-Dextran. The protein (b) and mRNA (c) levels of permeability-related factors including VE-cadherin, ZO-1, Claudin 5 and Occludin. ***P < 0.001 vs. control; ^###^P < 0.001 vs. LPS. ^$^P < 0.05 and ^$$$^P < 0.001 vs. LPS + si-RIP140 + Ov-NC.

To further demonstrate the interrelationship between CTBP2 and RIP140, CTBP2 was overexpressed by transfection of Ov-CTBP2 (Figures 5a-5b). Subsequently, the inflammatory response, apoptosis and permeability of HPMECs were respectively detected again. It was noticed that si-RIP140 could inhibit TNF-α, IL-1β and IL-6 expression compared with LPS-induced group, but the presence of Ov-CTBP2 significantly reversed the inhibitory effect of si-RIP140 (Figure 5c). Also, Western blotting analysis showed that overexpression of CTBP2 could significantly abrogate the inhibitor effect of RIP140 on the protein levels of COX2 and p/t-NF-κB p65 (Figure 5d). Similarly, TUNEL assay showed that the apoptosis in the LPS + si-RIP140 + Ov-CTBP2 group was notably promoted compared to LPS + si-RIP140 + Ov-NC (Figure 6a). Moreover, the effect of si-RIP140 on the expression of Bcl2, Bax and cleaved caspase3 was reversed by CTBP2 overexpression (Figure 6b). The experimental results from FITC-dextran and Western blotting showed that overexpression of CTBP2 not only reversed the inhibitory effect of si-RIP140 on the permeability of HPMECs, but also rescued the enhanced expression of permeability-related proteins imposed by RIP140 depletion (Figure 7a-7c).

## Discussion

ALI and acute respiratory distress syndrome (ARDS) are characterized by excessive inflammatory response of lung tissues, disruption of the functional barrier and pulmonary edema which results in severe impaired gas exchange [16]. Meanwhile, they are extremely common in pediatric intensive care cases [17,18]. RIP140, as an important transcriptional co-repressor, could play an important role in energy metabolism and reproduction by interacting with different nuclear receptors [19,20]. Recent studies have revealed that RIP140 could also be involved in the regulation of inflammatory responses by enhancing the transcriptional activity of NF-κB and inhibiting the functional activity of anti-inflammatory nuclear receptors [21,22]. In this research, through database prediction and Co-IP validation, the correlation of RIP140 and CTBP2 was successfully demonstrated for the first time.

In the early stage of ALI/ARDS, the inflammatory response of macrophages is activated by various pathogenic factors, which immediately releases a series of key pro-inflammatory factors, such as TNF-α, IL-1β, and IL-6 [23]. This was also demonstrated by markedly higher levels of TNF-α, IL-1β and IL-6 expression in HPMECs following LPS induction compared to the control group in the present study. Meanwhile, the expression levels of inflammation-related factors COX2 and NF-κB p65 were also significantly increased in HPMECs exposed to LPS. It was noteworthy that the expression of inflammatory factors and inflammation-related factors was suppressed after RIP140 was silenced in this study, compared to the LPS-induced group. Nowadays, CTBP2 has been reported to regulate the activation of LPS-induced microglia cells and to have an important role in the immune response [24]. Furthermore, inhibition of CTBP was found to potentially reduce inflammation and neurological impairment in a study of a mouse model of traumatic brain injury [25]. Likewise, in our study, the inhibitory effect of RIP140 interference on inflammation production was obviously reversed after CTBP2 overexpression. Such results not only demonstrated that CTBP2 overexpression promoted LPS-induced inflammation in HPMECs, but also proved that RIP140 might inhibit LPS-induced LPS-induced inflammatory of HPMECs by regulating CTBP2.

In addition, cell apoptosis is one of the main causes of ALI [26,27]. The balance of endothelial cell (EC) survival and death is critical for angiogenesis, vascular atrophy, and barrier function [28,29]. Due to the unique location at the interface of circulating blood and surrounding tissues, EC may be exposed to a variety of environmental stresses or internal stresses [27]. Thus, as a pathophysiological consequence of these stimuli, apoptosis is essential for the investigation of lung diseases. In the present study, LPS induction remarkably increased the apoptosis level of HPMECs, while the analysis of apoptosis-related proteins also indicated the change of apoptosis level. In turn, after RIP140 was inhibited, the apoptosis level of LPS-induced HPMECs was suppressed. For the mechanism, CTBP2 acts as a transcriptional co-repressor and is involved in cell migration, apoptosis and tumor formation [30]. The inhibitory effect of si-RIP140 on LPS-induced apoptosis in HPMECs was reversed after CTBP2 overexpression. This demonstrated that RIP140 mediated CTBP2 to regulate the apoptosis of LPS-induced HPMECs.

More importantly, ALI/ARDS is an acute episode of non-cardiogenic pulmonary edema caused by increased pulmonary vascular permeability [31]. Therefore, cellular permeability is another important indicator for the assessment of lung injury. Dong et al. found a significant increase in EC permeability after LPS induction in their study, which might disrupt cellular integrity [32]. This finding was also verified in the present study. Moreover, the current study implied that the permeability of LPS-induced HPMECs was regulated and restored by downregulating RIP140. Subsequently, upregulation of CTBP2 was investigated on the mechanism and the results clearly showed that the effects produced by RIP140 depletion could be reversed by overexpression of CTBP2, further demonstrating that RIP140 knockdown mitigated cell permeability by regulating CTBP2.

## Conclusion

Taken together, these results indicated for the first time that downregulation of RIP140 could alleviate LPS-induced inflammation, apoptosis and permeability in HPMECs by regulating CTBP2. The current study focused on the analysis in cellular models. Considering the limitations of the study, further validation of the results of this study in animal models will follow. It might be expected to provide new insights into the treatment of lung injury in children.

## Highlights

(1) Knockdown of RIP140 inhibited LPS-induced inflammatory response in HPMECs.

(2) Knockdown of RIP140 reduced LPS-induced permeability in HPMECs.

(3) CTBP2 could bind to RIP140.

(4) Interfering with RIP140 reduces CTBP2 to attenuate LPS-induced HPMEC damage.
